# Variations of Bacterial and Diazotrophic Community Assemblies throughout the Soil Profile in Distinct Paddy Soil Types and Their Contributions to Soil Functionality

**DOI:** 10.1128/msystems.01047-21

**Published:** 2022-03-01

**Authors:** Xiaomi Wang, Ying Teng, Wenjie Ren, Yuntao Li, Teng Yang, Yan Chen, Ling Zhao, Huimin Zhang, Eiko E. Kuramae

**Affiliations:** a Key Laboratory of Soil Environment and Pollution Remediation, Institute of Soil Science, Chinese Academy of Sciences, Nanjing, People’s Republic of China; b Shanghai Majorbio Bio-pharm Biotechnology Co., Ltd., Shanghai, People’s Republic of China; c Department of Microbial Ecology, Netherlands Institute of Ecology (NIOO-KNAW), Wageningen, Netherlands; d Institute of Environmental Biology, Ecology and Biodiversity, Utrecht University, Utrecht, Netherlands; California Department of Water Resources

**Keywords:** hydragric anthrosols, soil horizon, bacterial community, diazotrophic community, nitrogen and iron cycling

## Abstract

Soil microbiota plays fundamental roles in maintaining ecosystem functions and services, including biogeochemical processes and plant productivity. Despite the ubiquity of soil microorganisms from the topsoil to deeper layers, their vertical distribution and contribution to element cycling in subsoils remain poorly understood. Here, nine soil profiles (0 to 135 cm) were collected at the local scale (within 300 km) from two canonical paddy soil types (Fe-accumuli and Hapli stagnic anthrosols), representing redoximorphic and oxidative soil types, respectively. Variations with depth in edaphic characteristics and soil bacterial and diazotrophic community assemblies and their associations with element cycling were explored. The results revealed that nitrogen and iron status were the most distinguishing edaphic characteristics of the two soil types throughout the soil profile. The acidic Fe-accumuli stagnic anthrosols were characterized by lower concentrations of free iron oxides and total iron in topsoil and ammonia in deeper layers compared with the Hapli stagnic anthrosols. The bacterial and diazotrophic community assemblies were mainly shaped by soil depth, followed by soil type. Random forest analysis revealed that nitrogen and iron cycling were strongly correlated in Fe-accumuli stagnic anthrosol, whereas in Hapli soil, available sulfur was the most important variable predicting both nitrogen and iron cycling. The distinctive biogeochemical processes could be explained by the differences in enrichment of microbial taxa between the two soil types. The main discriminant clades were the iron-oxidizing denitrifier *Rhodanobacter*, *Actinobacteria*, and diazotrophic taxa (iron-reducing *Geobacter*, *Nitrospirillum*, and *Burkholderia*) in Fe-accumuli stagnic anthrosol and the sulfur-reducing diazotroph *Desulfobacca* in Hapli stagnic anthrosol.

**IMPORTANCE** Rice paddy ecosystems support nearly half of the global population and harbor remarkably diverse microbiomes and functions in a variety of soil types. Diazotrophs provide significant bioavailable nitrogen in paddy soil, priming nitrogen transformation and other biogeochemical processes. This study provides a novel perspective on the vertical distribution of bacterial and diazotrophic communities in two hydragric anthrosols. Microbiome analysis revealed divergent biogeochemical processes in the two paddy soil types, with a dominance of nitrogen-iron cycling processes in Fe-accumuli stagnic anthrosol and sulfur-nitrogen-iron coupling in Hapli stagnic anthrosol. This study advances our understanding of the multiple significant roles played by soil microorganisms, especially diazotrophs, in biogeochemical element cycles, which have important ecological and biogeochemical ramifications.

## INTRODUCTION

Paddy fields are critical for agricultural production worldwide and support more than 50% of the world’s population (1). During periodic flooding-drying water management, paddy soils undergo dramatic vertical shifts in environmental conditions (e.g., moisture, oxygen, redox conditions, and resource availability) (1–3). The soil microbiotas inhabiting paddy soils are key drivers and indicators of multiple soil ecosystem functions throughout the soil profile, including biogeochemical element cycling, soil formation, plant nutrient provision, and pollutant degradation (4, 5). Although most studies have focused exclusively on the top 40 cm of soil (3, 6, 7), the subsoil harbors more than two-thirds of the total soil nutrient pool and 35 to 50% of soil microbial biomass (8, 9). The subsoil microbiota in terrestrial ecosystems (e.g., arable, forest, and karst soils) possesses intense activity (10) and differs from those in the topsoil (8–13). Paddy soils represent an intermediate system between terrestrial ecosystems and aquatic ecosystems, and the depletion of oxygen in paddy soil after flooding leads to the prevalence of anaerobic microbial groups with specific functions (functional microorganisms), including nitrate reducers, iron reducers, sulfate reducers, and methanogens (14). However, information on the shifts in the microbial community with depth in paddy soils and their functional potential is limited.

The soil profile reflects the specific developmental history of the soil and is ideal for exploring the unique microbial communities in subsoils (15, 16). As a result of soil-forming factors (e.g., climate, parent material, organisms, and time) and management practices, soil systems gradually develop into distinct genetic horizons throughout the soil profile, leading to vertical stratifications of soil physicochemical properties (16). As for rice production, soil texture and nutrient availability along soil profile have been shown to influence root morphology and total nutrient uptake, thereby determining crop productivity (17). Some metallic elements (e.g., Fe and Mn) accumulated around roots could facilitate the exclusion of heavy metal uptake, thereby reducing phytotoxicity (18). Soil microbial community structure and functional profile could be also horizon-specific (16), but the majority of studies of microbial distribution artificially separate the soil profile into uniform depths (layers of 10 to 20 cm) (19–21), overlooking the discontinuity and inhomogeneity among distinct layers (16). Application of sampling increments based on distinct genetic horizons along the soil profile can provide statistically significant resolution for the analysis of edaphic physicochemical stratification and microbial responses with depth (22). Analyzing the genetic features (e.g., soil color and edaphic characteristics) of distinct horizons throughout the soil profile for soil stratification, which is generally applied for soil diagnostics and classification, would provide high resolution for studying depth-related changes in soil microbial community structure and their associations with element biotransformation (16, 23).

Soil microbial distribution patterns are also influenced by soil type (24, 25). Paddy soils evolve into a variety of types depending on topography, flooding history, groundwater table, and parent materials (15). Based on soil moisture regimes and associated redoximorphic features, as revealed by the genetic horizons across the soil profile, paddy soils can be grouped into three types: oxidizing, redoximorphic, and reducing (15). Redoximorphic soils are characterized by both reducing and oxidizing environments throughout the soil profile and usually have a history of long-term flooding and seasonal fluctuations of groundwater. Oxidative soils are dominated by an oxidizing environment in the soil profile and typically feature short-term flooding and deep groundwater. Edaphic morphological and physicochemical properties throughout the soil profile vary significantly among different paddy soil types, including differences in soil pH, redox potential, and the accumulation of specific elements (e.g., iron [Fe] and manganese [Mn]) (15). These differences are expected to be highly correlated with the structure and functioning of the soil microbiome (24). Compared with the plough layer, which is highly modified by anthropogenic management, the edaphic properties and microbial activity in subsurface horizons are more sensitive to soil type (15). The few studies of the temporal distributions of the bacterial and fungal communities in different types of paddy soil have selected sampling sites across large geographical distances (>1,000 km between sites) (19, 21, 26); thus, the results might be significantly influenced by distinct environmental variables (e.g., temperature and precipitation). Studies at a local scale would more precisely reflect the associations between microbiota and edaphic features throughout the profiles of different soil types.

In addition, examining functional microbiomes, such as the diazotrophic community, would improve the accuracy and resolution of assessments of microbial community function (27). Nitrogen (N_2_)-fixing microorganisms are the predominant source of active N in the biosphere (∼50 to 70 Tg N year^−1^ to agricultural systems) (28, 29). As a key driver of global N dynamics, versatile diazotrophs also participate in other major biogeochemical cycles, such as carbon sequestration and Fe and sulfur (S) cycling (14, 28, 30–35). However, studies of the biogeographic patterns of diazotrophic communities in paddy soils in recent decades have focused only on topsoil (6, 25). Studies showed that *Cyanobacteria* and *Proteobacteria* were the dominant diazotrophs and are critical for N_2_-fixing activity in the superficial layer of paddy soils (8, 36, 37). However, due to vertical changes in soil conditions (e.g., pH, redox potential [Eh], oxygen, and light), the composition and activity of the diazotrophic community may vary throughout the soil profile (13). Therefore, deeper exploration of the diazotrophic community assembly in the deep layers of paddy soils and its contribution to element biogeochemical cycles in different soil types is needed.

**FIG 1:**
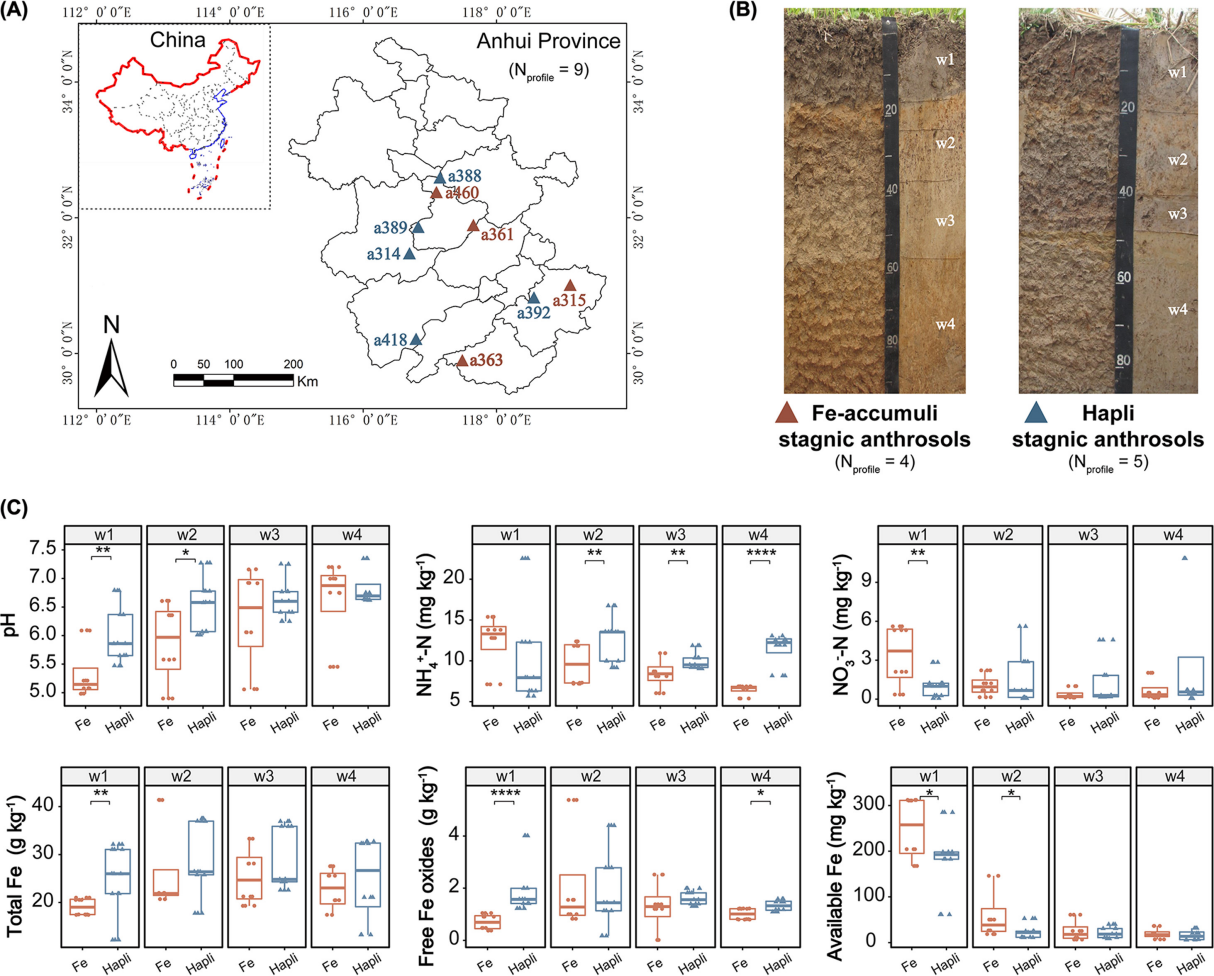
(A) Location of paddy soil sampling sites (Anhui Province, China). The maps are from the DataV.GeoAtlas data visualization platform and use data from the Autonavi open platform. (B) Representative soil profiles of Fe-accumuli and Hapli stagnic anthrosols. (C) Variations in the main differentiated edaphic properties (soil pH, N, and Fe) in the two typical paddy soil types throughout the soil profiles. Significant differences were evaluated according to Mann-Whitney nonparametric tests (*, *P < *0.05; **, *P < *0.01; ****, *P < *0.0001). Data from the fifth soil layers (w5) are not shown because only two profiles (a363 and a418) possessed w5.

The community structure of soil microbiota is closely linked to biogeochemical element cycling, soil formation, and crop productivity, but the response of the microbial community, especially N_2_-fixing microorganisms, to soil depth and types in paddy soils remains largely unknown. In this study, paddy soils in Anhui Province, a major rice production area in China (38), were sampled at nine sites separated by a maximum distance of 300 km to minimize the effects of spatial distance and climate on edaphic properties and the microbial community (Fig. 1A). At each site, one soil profile (0 to 135 cm) was excavated. According to the redoximorphic features and degree of paddy soil development indicated by the genetic horizons, two canonical paddy soil types were classified: (i) Fe-accumuli anthrosol, a redoximorphic type, and (ii) Hapli stagnic anthrosol, an oxidative type (15, 39, 40). The abundance and structure of the bacterial and diazotrophic communities at each horizon were assessed using quantitative PCR (qPCR) and Illumina MiSeq sequencing. We posited that (i) both paddy soil type and depth affect edaphic characteristics and microbial community assembly and (ii) variations in microbial community assembly throughout the soil profile are correlated with differences in soil functioning between the two paddy soil types.

## RESULTS

### Edaphic characteristics throughout the profiles of the two paddy soil types.

Throughout the profiles of the sampled paddy soils, obvious stratification of the soil was noted (Fig. 1B). At each genetic horizon, the morphological characteristics and edaphic properties differed greatly between the two soil types (see Fig. S1 in the supplemental material). In general, soil pH increased markedly and approached 7 with increasing soil depth at all sampled sites. Compared with the topsoil (w1), the deeper layers (w2 to w4) harbored significantly lower concentrations of some nutrients (*P < *0.01), such as organic matter (OM), N (total N, available N, and NH_4_^+^-N), P (total and available P), Fe (total and available Fe), and Mn (total and available Mn). In contrast, K, Ca, and total Mg/Cu/Zn did not differ significantly with soil depth (Fig. S1).

10.1128/mSystems.01047-21.1 FIG S1 Soil physiochemical properties of the two paddy soil types at each soil depth. w1 to w4 indicate different soil layers. Significant difference was evaluated according to the Mann-Whitney nonparametric tests (*, *P* < 0.05; **, *P < *0.01; ***, *P < *0.001; insignificant levels are not shown). Download FIG S1, PDF file, 0.4 MB. Copyright © 2022 Wang et al. 2022 Wang et al. https://creativecommons.org/licenses/by/4.0/ This content is distributed under the terms of the Creative Commons Attribution 4.0 International license.

Soil pH, Fe, and N were the most differentiated edaphic properties between the two paddy types (Fig. 1C and Fig. S1). The pH in the upper layers (w1 and w2) was significantly lower (4.90 to 6.61) in Fe-accumuli than Hapli (pH 5.47 to 7.27) (*P < *0.05) stagnic anthrosol, whereas the pH in the deep layers (w3 and w4) was not significantly different between the two soil types (*P > *0.05). The accumulation of reddish Fe oxides (Fe plaque) in the subsurface layer (hydragric horizon) is a diagnostic feature of Fe-accumuli soil (Fig. 1B), and for this soil type the levels of free Fe oxides (>1.5-fold) and total Fe were higher in the subsoil (w2) than in the surface horizon (w1) (Fig. 1C). In contrast, available Fe tended to be enriched in the superficial layers of Fe-accumuli, and the concentration of available Fe was significantly higher in this soil type than in the topsoil of Hapli (*P < *0.05). In terms of N status, Fe-accumuli appeared to be depleted in total N and NH_4_^+^-N in deep layers (w3 and w2 to w4, respectively), the levels of which differed significantly (*P < *0.05) between the two soil types. These edaphic differentiations were also confirmed by random forest (RF) classification analysis, which revealed that soil type was best distinguished by free Fe oxides in topsoil and NH_4_^+^-N in deep layers (w2 to w4) (Fig. S2).

10.1128/mSystems.01047-21.2 FIG S2 Random forest classification model showed the mean predictor importance (mean decrease in accuracy) of spatial distance, climate, edaphic properties, and soil multielement cycling indices as drivers for the soil classification in the superficial (w1), the second (w2), and the deep layers (w3 and w4). The most differentiated factors are highlighted in boldface (red). *, *P < *0.05; **, *P < *0.01. N and Fe cycling indices were calculated by normalizing and standardizing each of the N/Fe-related nutrient properties. Download FIG S2, PDF file, 0.2 MB. Copyright © 2022 Wang et al. 2022 Wang et al. https://creativecommons.org/licenses/by/4.0/ This content is distributed under the terms of the Creative Commons Attribution 4.0 International license.

### Divergent assembly patterns of microbial communities throughout the profiles of the two soil types.

**FIG 2:**
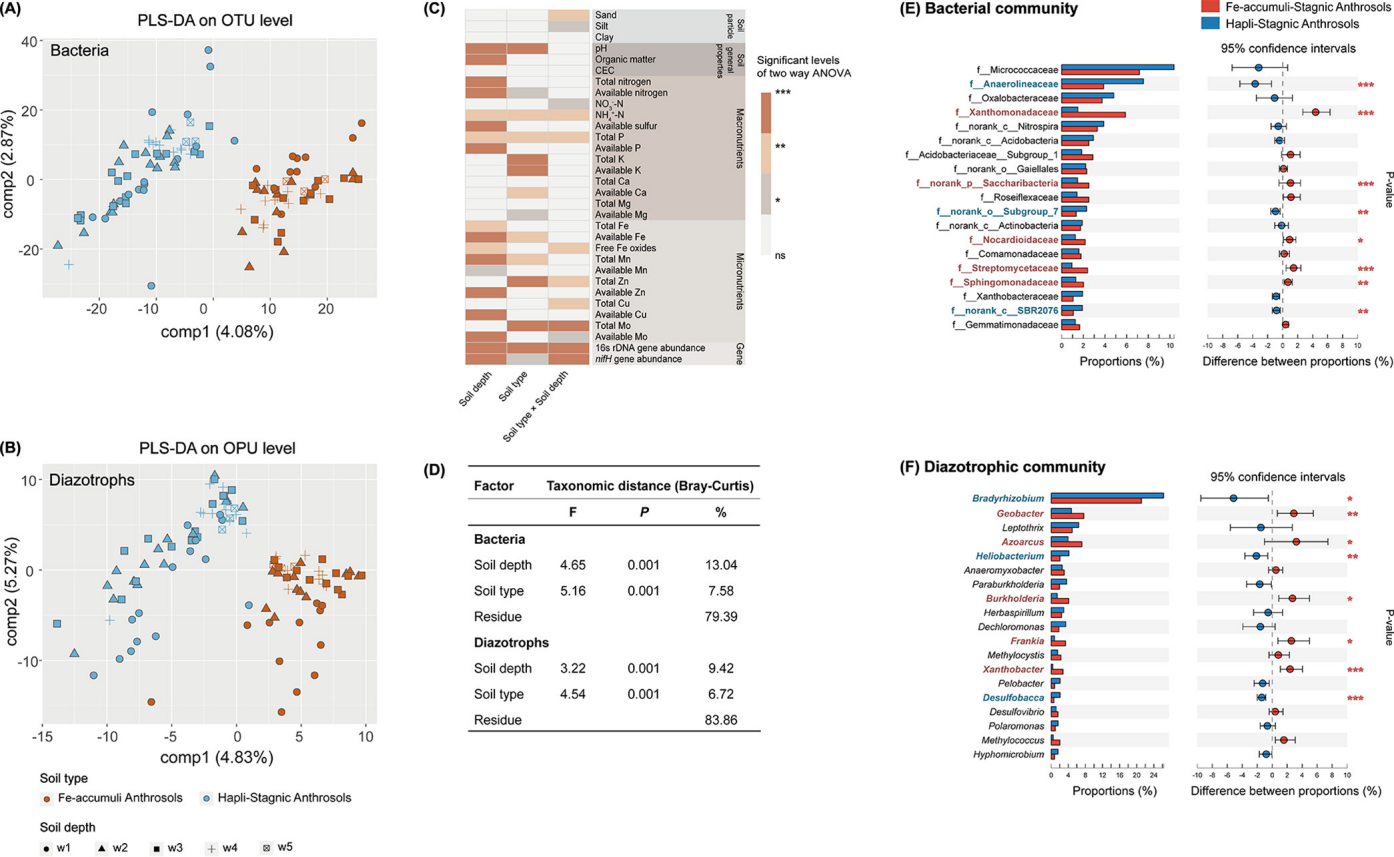
(A and B) Partial least-squares discriminant analysis (PLS-DA) plots showing the turnover of bacterial and diazotrophic community structures based on Bray-Curtis distances. (C and D) Bacterial and diazotrophic community compositions associated with the two types of paddy soils at the family and genus levels, respectively. Asterisks indicate significant differences according to Mann-Whitney nonparametric tests (FDR-corrected *P* value). (E) Impact of soil depth and type on edaphic variables and functional gene abundances determined by two-way ANOVA. (F) Factors determining the variation in bacterial and diazotrophic communities using PERMANOVA (999 permutations) of Bray-Curtis dissimilarity distances for the indicated factors. In each analysis, *F*, the *P* value, and the percentage of variation (%) explained by each factor refer to the total variance reported. ***, *P < *0.001; **, *P < *0.01; *, *P < *0.05; ns, not significant.

The vertical assembly patterns of the total bacterial and diazotrophic communities in the two soil types were analyzed. As soil depth increased, bacterial and diazotrophic α-diversity indices, including observed operational taxonomic unit (OTU) (Sobs; richness) and Shannon (taxonomic diversity) indices, displayed decreasing trends (Fig. S3), consistent with the patterns of the gene abundances of total bacteria (16S rRNA) and *nifH* genes quantified by qPCR (Fig. S3). However, microbial diversity and abundance did not differ significantly (*P > *0.05) between the two soil types, except for significantly higher bacterial and diazotrophic abundances in the w2 layer of Fe-accumuli. In partial least-squares discriminant analysis (PLS-DA), soil samples from Fe-accumuli and Hapli formed distinct clusters (Fig. 2A and B). Analysis of similarities (ANOSIM) and permutational multivariate analysis of variance (PERMANOVA or ADONIS) analysis further confirmed shifts in the structures of the bacterial and diazotrophic communities with both soil depth and type, with statistically significant differences at the OTU/operational protein unit (OPU) level (*P ≤ *0.001) (data not shown).

10.1128/mSystems.01047-21.3 FIG S3 Variations of bacterial and diazotrophic α-diversity between the two paddy soil types based on Sobs (observed operational taxonomic units) (A and B), Shannon index (C and D), and microbial gene copy numbers (E and F). Significant difference was evaluated according to Mann-Whitney nonparametric tests (*, *P* < 0.05; **, *P < *0.01; insignificant levels are not shown). w1 to w4 indicate different soil layers. Significant difference was evaluated according to the Mann-Whitney nonparametric test (*, *P* < 0.05; **, *P < *0.01; ***, *P < *0.001). Download FIG S3, PDF file, 0.5 MB. Copyright © 2022 Wang et al. 2022 Wang et al. https://creativecommons.org/licenses/by/4.0/ This content is distributed under the terms of the Creative Commons Attribution 4.0 International license.

### Environmental factors shape microbial assembly.

Applying the partial Mantel test to whole layers between soil types revealed that the diazotrophic community was more sensitive to geographic distances (*R* = 0.28 to 0.32) than to environmental heterogeneity (*R* = 0.13 to 0.19), whereas geographic and environmental variables contributed relatively equally to bacterial community dissimilarity (Table S2). These data were further confirmed by distance-decay patterns (Fig. S4). Environmental and geographic distances exerted greater impacts on diazotrophic community dissimilarities in Fe-accumuli (*R* = 0.19 and 0.32, respectively) than in Hapli (*R* = 0.13 and 0.28, respectively). Similar trends were observed for the bacterial community.

10.1128/mSystems.01047-21.4 FIG S4 Relationships between bacterial and diazotrophic community similarities and geographic distance (A and C) and environmental dissimilarity (B and D) in the paddy soil samples from two soil types. The geographic distance was determined in meters, environmental dissimilarity was based on Euclidean, and microbial community similarity was based on Bray-Curtis distance. Download FIG S4, PDF file, 0.3 MB. Copyright © 2022 Wang et al. 2022 Wang et al. https://creativecommons.org/licenses/by/4.0/ This content is distributed under the terms of the Creative Commons Attribution 4.0 International license.

10.1128/mSystems.01047-21.10 TABLE S2 Partial Mantel tests to compare relative impacts of environmental heterogeneity/dissimilarity (Euclidean distance) and geographical distance (Bray-Curtis distance) on microbial community. Microbial compositional dissimilarity was calculated in Bray-Curtis distance. Download Table S2, XLSX file, 0.01 MB. Copyright © 2022 Wang et al. 2022 Wang et al. https://creativecommons.org/licenses/by/4.0/ This content is distributed under the terms of the Creative Commons Attribution 4.0 International license.

The Mantel test further showed that the structures of the bacterial and diazotrophic communities were significantly and strongly correlated with edaphic properties (e.g., soil pH and available Mg, Ca, N, and Fe), except for soil texture (clay, sand, and silt), total Mg, and total Mo (Fig. S5). Comparison of the soil types revealed more significant correlations between microbial community assembly and edaphic factors in Fe-accumuli than in Hapli. The three most differentiated indices (NH_4_^+^-N, available Fe, and free Fe oxides) between the two soil types were significantly correlated with bacterial and diazotrophic community structures in Fe-accumuli (*P < *0.05) but not Hapli (*P > *0.05), except for an obvious effect of available Fe on the diazotrophic community in Hapli (*P < *0.05).

10.1128/mSystems.01047-21.5 FIG S5 Environmental drivers of bacterial and diazotrophic community compositions in Fe-accumuli (A) and Hapli stagnic anthrosols (B). Pairwise comparisons of environmental factors are shown, with a color gradient denoting Spearman’s correlation coefficients (*, *P < *0.05; **, *P < *0.01; ***, *P < *0.001). Taxonomic community composition was related to each environmental factor by Mantel tests. Edge width corresponds to the Mantel’s *r* statistic for the corresponding distance correlations, and edge color denotes the statistical significance based on 9,999 permutations. Download FIG S5, PDF file, 0.5 MB. Copyright © 2022 Wang et al. 2022 Wang et al. https://creativecommons.org/licenses/by/4.0/ This content is distributed under the terms of the Creative Commons Attribution 4.0 International license.

### Impacts of soil depth and type on edaphic properties and microbial community assembly.

To evaluate the impacts of soil depth and type on edaphic properties, two-way analysis of variance (PERMANOVA) was applied (Fig. 2C). Soil pH, N status (available N and NH_4_^+^-N), available Fe, total P, and total Mn were significantly influenced by both soil depth and type (*P < *0.05). OM, total N, total Fe, and available elements (S, P, Mn, Zn, and Cu) were significantly influenced only by soil depth (*P < *0.05), whereas significant impacts of soil type were restricted to K content and some micronutrients (total Zn and total Mo, available Ca and available Mg) (*P < *0.05).

PERMANOVA also confirmed that the microbial populations were significantly sensitive to both soil depth and type (Fig. 2D). Soil depth was the major determinant of bacterial and diazotrophic community structure separation, explaining 13.04% and 9.42% of the observed structure variation (*P = *0.001), respectively, followed by soil type (7.58% and 6.72%, *P = *0.001).

### Identification of discriminant microbial taxa characterizing soil types.

Throughout the entire soil profile, *Micrococcaceae*, *Anaerolineaceae*, *Oxalobacteraceae*, *Xanthomonadaceae*, and norank taxa belonging to *Nitrospira* were the dominant bacterial families, accounting for 21.72% and 28.84% of abundance in Fe-accumuli and Hapli, respectively (Fig. 2E). In the diazotrophic community, *Bradyrhizobium* was the major genus (21.11 and 26.27% in Fe-accumuli and Hapli, respectively), followed by *Geobacter* (7.63 and 4.75%), *Azoarcus* (7.18 and 3.96%), *Leptothrix* (4.90 and 6.42%), and *Heliobacterium* (2.06 and 4.16%) (Fig. 2F).

Among the significantly differentiated bacterial taxa between the two soil types (at the family level, *P < *0.05; Fig. S6A), *Acidobacteriaceae* subgroup 1 was enriched in the topsoil (w1) of Fe-accumuli. *Xanthomonadaceae* (especially *Rhodanobacter*, which was enriched in all layers), norank family, belonging to *Saccharibacteria* (w3), *Actinobacteria* (*Nocardioidaceae* and *Streptomycetaceae* in w2 and w3), and *Sphingomonadaceae* (w4), were characteristic of Fe-accumuli. Among the classified bacterial taxa in Hapli, *Xanthobacteraceae* and *Oxalobacteraceae* (especially *Massilia*) were enriched in the topsoil (w1), while *Anaerolineaceae* was enriched in the subsurface layers (w2 to w4).

10.1128/mSystems.01047-21.6 FIG S6 Discriminant bacterial (A, at family or genus level) and diazotrophic (B, at genus level) taxa across soil profiles or between Fe-accumuli and Hapli stagnic anthrosols across the soil profiles. Significant difference was evaluated according to the Kruskal-Wallis H tests among varied soil depths or Mann-Whitney nonparametric tests between two groups (*, *P* < 0.05). Only the differentiated taxa with significant levels are shown here. Download FIG S6, TIF file, 1.5 MB. Copyright © 2022 Wang et al. 2022 Wang et al. https://creativecommons.org/licenses/by/4.0/ This content is distributed under the terms of the Creative Commons Attribution 4.0 International license.

Among diazotrophs (Fig. S6B), the differentiated taxa in Fe-accumuli accounted for 70 to 80% of all the discriminant genera, including *Geobacter* (w2 and w4) and aerobic diazotrophs in the deep layers (*Azoarcus*, *Burkholderia*, and *Nitrospirillum* in w3 and w4, *Frankia* and *Xanthobacter* in w3). Hapli was characterized by *Bradyrhizobium*, *Heliobacterium* (w2), and anaerobic sulfur-reducing bacteria (SRB), including *Desulfobacca* (w1 to w3), *Desulfovibrio* (w3), and *Desulfomonile* (w4). The discriminant microbial taxa were also confirmed by random forest (RF) classification analysis, which showed that soil type could be correctly predicted by 81.50 to 96.30% of the variations in microbial composition, with higher assessment accuracy for bacterial predictors than for diazotrophic genera (Fig. S7).

10.1128/mSystems.01047-21.7 FIG S7 Discriminant bacterial (A) and diazotrophic (B) genera characterizing Fe-accumuli and Hapli stagnic anthrosol, respectively, across soil profiles analyzed by random forest classification analysis. OOB error indicates out-of-bag error. Red dots indicate Fe-accumuli type soils, and blue dots indicate Hapli-type soils. Download FIG S7, TIF file, 1.2 MB. Copyright © 2022 Wang et al. 2022 Wang et al. https://creativecommons.org/licenses/by/4.0/ This content is distributed under the terms of the Creative Commons Attribution 4.0 International license.

### Contributions of edaphic and microbial variables to the differentiation of soil N and Fe cycling processes.

**FIG 3:**
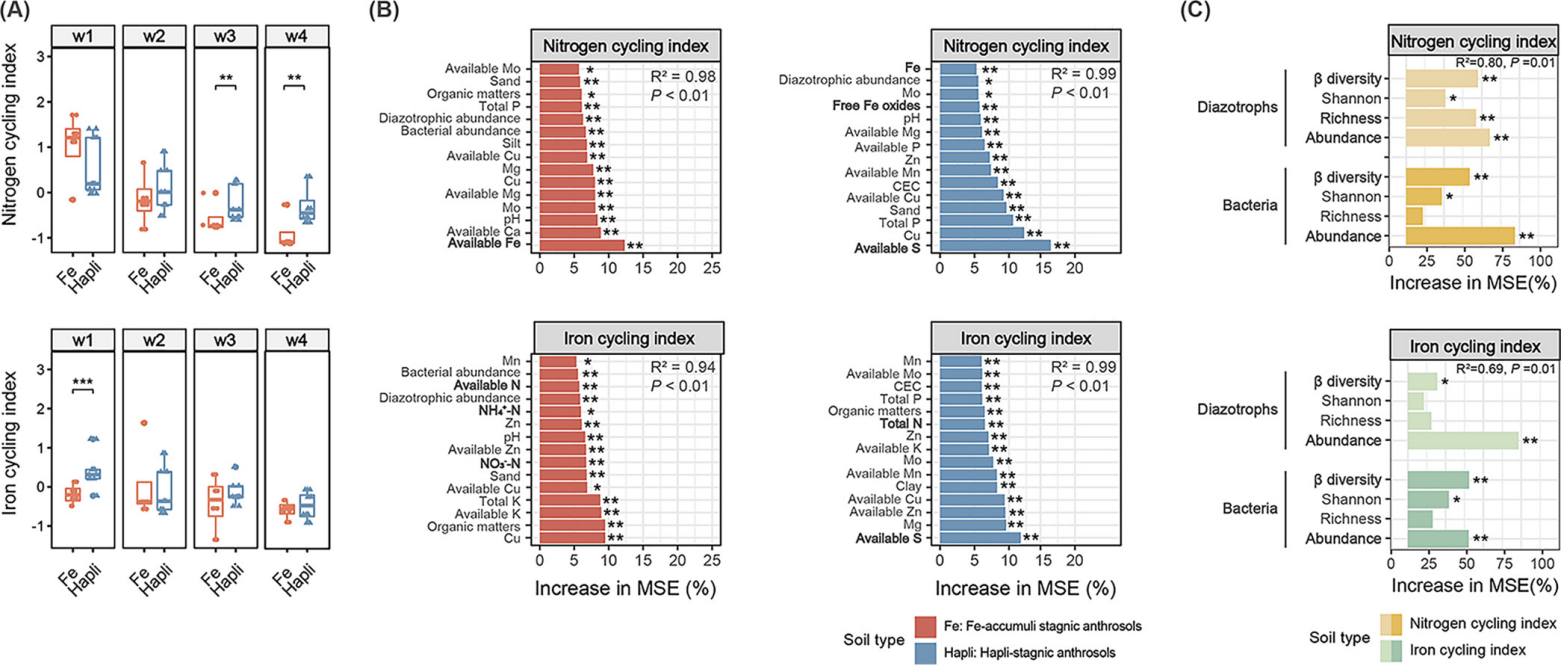
(A) Average nitrogen (N) and iron (Fe) cycling indices in the two paddy soil types throughout the soil profiles. Significant differences were evaluated according to Mann-Whitney nonparametric tests. (B and C) Random forest regression models. (B) Mean predictor importance (increase in MSE%; percentage increase in mean square error) of edaphic properties and microbial factors as explanatory variables for the soil N and Fe indices of the two soil types in the whole profile. Only the top 15 significant variables are shown. Elements related to the coupling of N-Fe-sulfur cycles are highlighted in boldface. (C) Potential microbial drivers (bacterial and diazotrophic abundance, α- and β-diversity indices) predicting the soil N and Fe cycling indices of the two soil types throughout the whole profile. Only significant predictors are shown. Gene abundances were quantified by qPCR with specific primers. Microbial richness is represented by Chao1. β-Diversity indicates the first principal coordinate of PLS-DA. Significance levels: *, *P < *0.05; **, *P < *0.01.

The potential contributions of edaphic properties and microbial variables (abundance, richness, Shannon index, and β-diversity) to the variations in soil N and Fe cycling processes between the two soil types were evaluated by RF analysis. To comprehensively evaluate N/Fe cycling processes (the most differentiated properties between the two soil types), N and Fe cycling indices were determined by normalizing and standardizing each of the N- or Fe-related nutrient properties for each horizon (Fig. 3A) (41). The variations in N and Fe cycling indices between the two soil types were strongly predicted by edaphic properties (*R*^2^ = 0.94 to 0.99, *P = *0.01; Fig. 3B) and by microbial variables (*R*^2^ = 0.69 to 0.80, *P = *0.01; Fig. 3C). In Fe-accumuli, available Fe was the most important variable for explaining the variation in the N cycling index (Fig. 3B). Cu was the top explanatory factor for the Fe cycling index, and N status (NO_3_-N, NH_4_^+^-N, and available N) also contributed significantly to the Fe cycling index in Fe-accumuli (*P < *0.05). In contrast, in Hapli, available S was the most important variable for predicting both the N and Fe cycling indices. Among microbial predictors, diazotrophic and bacterial abundances best predicted the dynamics of the soil N and Fe cycling indices (*P < *0.01), followed by microbial β-diversity (*P < *0.05) (Fig. 3C).

**FIG 4:**
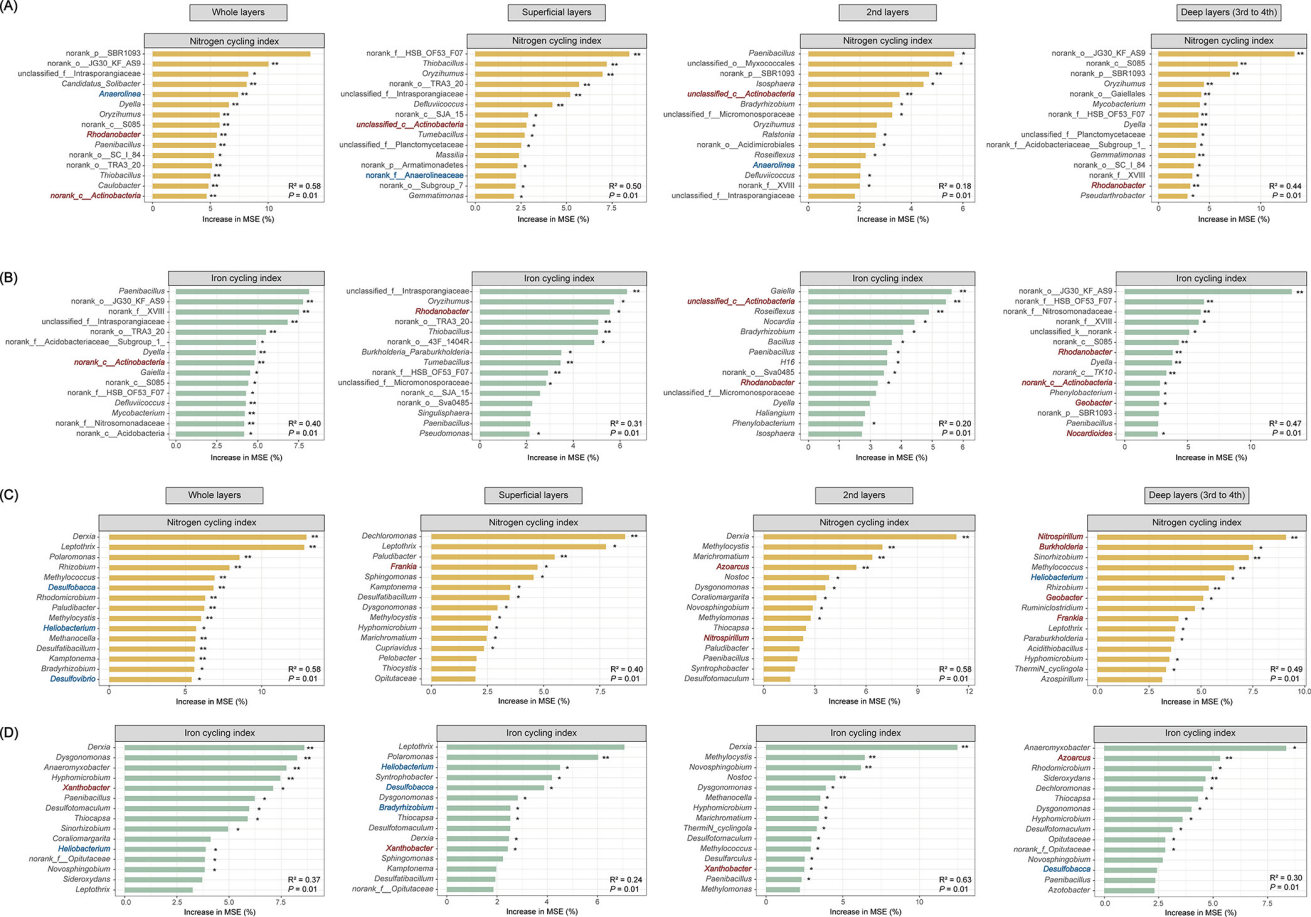
Potential bacterial (A and B) and diazotrophic drivers (C and D) predicting soil nitrogen (N) and iron (Fe) cycling indices in the whole profile and the superficial, second (2nd), and deep layers (3rd to 4th), respectively, as revealed by random forest analysis. *, *P < *0.05; **, *P < *0.01. Genera that are highlighted in red boldface are enriched in Fe-accumuli stagnic anthrosol, while taxa that are highlighted in blue boldface are abundant in Hapli stagnic anthrosol.

To evaluate the potential functions of the discriminant microbial taxa in the two soil types, the potential bacterial and diazotrophic drivers predicting the soil N and Fe cycling indices were determined by RF analysis. The microbial predictors of soil N and Fe cycling varied greatly among distinct soil horizons (Fig. 4). Among the discriminant bacterial genera in Fe-accumuli, *Rhodanobacter*, which was enriched throughout the soil profile, was important for predicting N cycling in the deep layers (w3 and w4) and Fe cycling in all layers (Fig. 4A and B). *Actinobacteria* (*Nocardioides* w3) were involved in soil N cycling in the upper layers (w1 and w2) and Fe cycling in the deep layers (w2 to w4, such as *Nocardioides*). Analysis of the Hapli samples indicated that taxa belonging to *Anaerolineaceae* (w2 to w4) (e.g., *Anaerolinea*) participate in soil N cycling in the upper layers (w1 and w2).

In the diazotrophic community, *Geobacter*, which was characteristic of Fe-accumuli, was a significant predictor of both N and Fe cycling in the deepest layers (w3 and w4; *P < *0.05) (Fig. 4B and C). The enriched aerobic diazotrophs in Fe-accumuli, especially *Burkholderia* and *Nitrospirillum*, were pivotal in predicting N cycling in the deep horizons (w3 and w4), whereas *Xanthobacter* (w3) explained the variation in the Fe cycling index (Fig. 4C to D). In Hapli, the anaerobic SRB *Desulfobacca* contributed significantly to soil N cycling throughout the whole profile and the Fe cycling index in the top (w1) and bottom horizons (w3 and w4) (Fig. 4D).

## DISCUSSION

**FIG 5:**
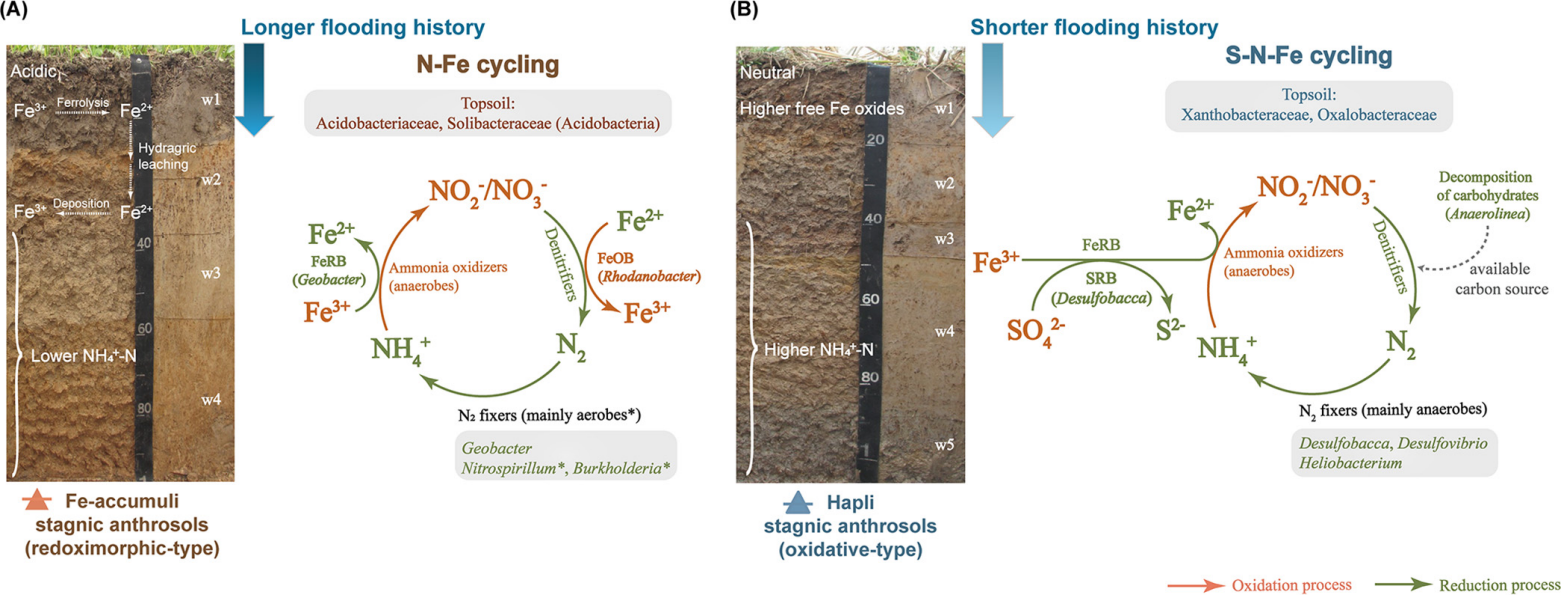
Proposed representative cycling processes of multiple elements (nitrogen, iron, and sulfur) in Fe-accumuli and Hapli stagnic anthrosols. FeRB, Fe^3+^-reducing bacteria; FeOB, Fe^2+^-oxidizing bacteria; SRB, sulfate-reducing bacteria; SOB, sulfur-oxidizing bacteria.

The subsoil ecosystem, consisting of complicated interplays among root, soil, and microorganisms, contributes greatly to the overall quality of soils (4). A better understanding of the microbial mechanisms driving soil quality and functioning is important for maintaining a productive soil environment for sustainable crop production (4). Clues from the soil profile can provide a detailed picture of what a soil is and does from the standpoint of soil development (42). In the present study, we found that soil nutrient status (especially the lower N and Fe cycling indices in Fe-accumuli soil) and microbial community assemblies in paddy soils varied significantly at the local scale depending on soil horizons, followed by soil types. In Fe-accumuli stagnic anthrosol, N and Fe cycling were strongly correlated (Fig. 3B) and may have been correlated with the main discriminant clades, the Fe-oxidizing denitrifier *Rhodanobacter*, and Fe-reducing diazotroph *Geobacter*. In contrast, in Hapli stagnic anthrosol, available S was the most important variable determining the N and Fe cycling indices, and turnover in S-N-Fe cycling may have been driven by the sulfur-reducing diazotroph *Desulfobacca*. Our results give distinct pictures of the divergent biogeochemical processes in the two paddy soil types that may be correlated with distinct microbial taxa (Fig. 5).

Our results showed that both soil depth and type influenced edaphic characteristics and microbial community assembly in the paddy soils, with a more significant impact of soil depth. In general, nutrient levels were higher in the topsoil than in deep layers, especially nutrients limiting for plant growth (e.g., OM, total N, NH_4_^+^-N, total P, available P, and available microelements), consistent with previous reports in dryland and paddy soils (7, 12, 19). Microbial abundance and diversity were also significantly higher in the upper layers (see Fig. S3 in the supplemental material), which could be related to a proper environment for microbial growth (e.g., light, oxygen, and enriched nutrients) in the plough layers (12). A few studies have reported decreases in diazotrophic abundance with depth (43, 44), but our data go further by providing a detailed representation of the changes in diazotrophic diversity and structure in subsoils, which differed significantly from those in topsoil (Fig. 2). However, the responses of microbial populations to soil depth depend on sampling site and microbial groups. Li et al. reported that at depths below 40 cm, the relative abundances of bacteria and fungi increased with depth at some sample sites, whereas the population of actinomycetes decreased (19). The different responses of distinct microbial groups to soil depth may reflect divergent requirements for nutrients and environmental factors such as oxygen, moisture, light, and pH (2, 45).

Paddy soil type was a secondary explanatory variable for the variations in edaphic factors and microbial community assembly. At each genetic horizon, microbial community abundance and diversity were generally consistent between the two paddy soil types (Fig. S3). In contrast, the structures of the bacterial and diazotrophic communities differed significantly, albeit slightly, between the two paddy soil types (Fig. 2), which could be attributed mainly to differences in the number of years of rice cultivation and the underground water table (15). The differences in fertilization practices in these sampling zones might also contribute to the dissimilarity in microbial community (3, 46). In contrast to the overwhelming contribution of soil depth to microbial community compared with soil type observed here, previous studies conducted at larger geographic scales have found a greater impact of soil type on microbiomes (6, 21, 24). Bai et al. showed that soil type exerted more significant impacts on bacterial and fungal diversity than soil depth in samples collected at large geographic distances (i.e., more than 1,000 km), and significant variations in functional structure were observed only among soil types and not among profile depths (21). Significant differences in topsoil diazotrophic communities between different soil types have also been observed in dryland but may reflect long distances between sampling sites and differences in climate and agricultural practices (25). In addition, previous studies have rarely separated soil horizons using genetic features, which might facilitate the identification of vertical changes in soil profiles. When combined with previous results, our data highlight the need to sample multilayered soil profiles, especially using genetic horizons, to comprehensively understand the structure and function of soil microbiomes.

The two representative paddy soil types identified here displayed divergent morphological and pedological features (especially for Fe and N contents) throughout the soil profile (Fig. 1B and C). Among Fe-related parameters, the topsoil of Fe-accumuli stagnic anthrosol tended to be bleaching, with 1.5 times fewer free Fe oxides than in the subsoil (Fig. 1C and Fig. S1) compared with the Hapli stagnic anthrosol, which had a shorter rice planting period (15, 39). The loss of Fe oxides in Fe-accumuli topsoil may be the result of intense ferrolysis (reduction of Fe^3+^ to Fe^2+^) and consequent reductive eluviation of more soluble Fe^2+^ under long-term anoxic waterlogged conditions (40), which might be related to the enrichment of *Geobacter* in this soil type (Fig. 4 and Fig. S6B and S7B). During the flooding of paddy soil, Fe^3+^ reduction is a typical terminal electron-accepting process at interfaces (e.g., water-soil and the rhizosphere), which is dominated by the Fe^3+^-reducing bacteria (FeRB) *Geobacter* and *Anaeromyxobacter*, as revealed by ^13^C-RNA-stable isotope probing (SIP) (1, 47). Deposition of extremely insoluble Fe/Mn oxides (Fe plaques) was observed in the hydragric horizon of Fe-accumuli soil (submerged type), imparting a reddish color (48), but not in well-drained soils (1). Fe plaques are considered to affect nutrient supply to the roots and, further, rice growth through influencing the transformation of multiple elements (e.g., carbon, N, and P) (1). For example, Fe plaque is believed to be a medium for rice P uptake, which significantly improves the P availability around the root surface (1). *Rhodanobacter*, which was enriched in Fe-accumuli, is known for its nitrate-reducing Fe^2+^-oxidizing ability under anaerobic conditions (49), which might explain the presence of Fe oxides and denitrification in this soil type. In addition, the acidic conditions (pH 5.34 ± 0.49) in the upper layers of Fe-accumuli soil might facilitate Fe activity and availability via Fe reduction (50). *Acidobacteriaceae*, which were enriched in Fe-accumuli topsoil (Fig. S6A), is commonly found in oligotrophic and low-pH conditions, such as paddy soils, forest soils, and especially acid mining areas (51–54).

The N cycling index was another edaphic factor that was differentiated between the two soil types. NH_4_^+^-N content decreased more sharply with depth in Fe-accumuli soil than in Hapli soil. Rice plant prefers NH_4_^+^-N to NO_3_^−^-N as an N source (1). For Hapli type soil, the greater supply of NH_4_^+^-N in the subsoils might contribute to root growth and plant nutrition through an enhanced photosynthesis rate (55). The lower NH_4_^+^-N storage in Fe-accumuli soil might be attributable to the reduced N input provided by diazotrophs and high N loss mediated by denitrification. In paddy soils, NH_4_^+^-N fixed by diazotrophs is the major source of reactive N for rice and heterotrophic organisms (29) and is also the most important factor shaping the diazotrophic community (56). The depletion of NH_4_^+^-N in Fe-accumuli subsoils might be attributable to the detrimental impact of acidic conditions in ferrosols on diazotrophic growth and nitrogenase activity (25). Previous studies have shown that N_2_-fixing ability is weaker in acidic soils than under neutral conditions (6). Notably, acidic Fe-accumuli tended to harbor high *nifH* abundance in the w2 layer (Fig. S3), consistent with previous studies, which might represent a compensation strategy of indigenous diazotrophs under N deficiency (6). Moreover, the relatively long-term flooding history of Fe-accumuli soil would be conducive to anoxic ammonia oxidation, nitrate leaching, and denitrification, thereby resulting in N loss, especially in the anaerobic deep layers (57, 58). In addition, Mo, a core element for the N cycle (e.g., N_2_ fixation, nitrification, denitrification, and dissimilatory nitrate reduction to ammonium) (59), was positively correlated with the N cycling index and diazotrophic community indices (Fig. 3B and Fig. S5). Mo deficiency-mediated suppression of N_2_ fixation capacity is a prevalent phenomenon in agricultural soils, with a threshold available Mo concentration of 0.15 mg kg^−1^ (37). In acidic Fe-accumuli, Fe and aluminum (Al) precipitation induce the formation of recalcitrant Mo (6, 50). The lower available Mo content in Fe-accumuli (0.02 ± 0.01 mg kg^−1^; Table S1) compared with Hapli might also limit nitrogenase activity and thereby impede the Mo-dependent N cycle.

10.1128/mSystems.01047-21.9 TABLE S1 Sampling site locations and physiochemical properties. Download Table S1, XLSX file, 0.04 MB. Copyright © 2022 Wang et al. 2022 Wang et al. https://creativecommons.org/licenses/by/4.0/ This content is distributed under the terms of the Creative Commons Attribution 4.0 International license.

In this study, the majority of diazotrophs discriminating the two soil types were aerobes that were enriched in Fe-accumuli (Fig. S7B). Some of these species are facultative anaerobes or perform microaerobic respiration, such as *Nitrospirillum* and *Burkholderia* (60, 61), which were critical for predicting N cycling in the deep layers of Fe-accumuli soil. The ability of these species to adapt to anoxic conditions might be related to the acid status and periodic redox alternation in this redoximorphic soil. Facultatively anaerobic diazotrophs reportedly can adapt to acidic conditions and act as dominant N_2_ fixers (62). The microaerobic respiration of diazotrophs could be more suitable for the functioning of oxygen-sensitive nitrogenase. Future studies could use ^15^N_2_-DNA-SIP to identify active N_2_ fixers in soils, such as cyanobacteria (mainly *Nostocales* and *Stigonematales*) in paddy topsoil (36). Combining DNA-SIP with nitrogenase activity analysis would further reveal whether these discriminant diazotrophs truly function in deep layers (6, 31).

Edaphic and microbial predictors (especially for the diazotrophic community) differed markedly between the two soil types, indicating distinctive element cycling processes. In Fe-accumuli, available Fe was the most important variable for controlling N cycling processes, and N pool status (NO_3_-N, NH_4_^+^-N, and available N) was strongly correlated with the Fe cycling index (Fig. 4B). Microbe-mediated coupling of the Fe and N cycles is typical of paddy soils (1). The two differentiated taxa in this soil type, *Geobacter* and *Rhodanobacter*, are expected to be related to coupling of N and Fe cycling. *Geobacter*, the diazotrophic keystone for N_2_ fixation in paddy soils (56), was identified as the discriminant taxon for Fe-accumuli (Fig. S6B and S7B) and was involved in both the Fe and N cycling indices (Fig. 4B and C). Anaerobically respiring *Geobacter* are a clade of active dissimilatory FeRB in rice paddy soil (47, 63). *Geobacter*-mediated Fe-ammox (iron reduction coupled to anaerobic ammonium oxidation) causes N loss from paddy soil (30), and stimulating Fe^3+^-reducing *Clostridiales* can improve the diazotrophic population in paddy soil (64), which might explain the elevated diazotrophic abundance in the subsurface of Fe-accumuli (Fig. S3). The denitrifying bacterial genus *Rhodanobacter* was another differentiated indicator of Fe-accumuli throughout the soil profile (Fig. S6B and S7B). The contribution of this anaerobic species to N and Fe cycling (Fig. 4A and B) might be due to its nitrate-dependent Fe-oxidizing capacity, which dominates in acidic nitrate-uranium-contaminated subsurface environments (65). Additionally, it is worth noting that microbial taxa present in a certain soil type not only exert impacts on soil nutrients but also continuously respond to changes of soil conditions (4). The structure and potential functioning of specific microbial taxa should be a result of the complex and dynamic interplays between microbial community and soil characteristics over time.

In Hapli, available S best predicted N and Fe cycling indices (Fig. 3B). Consistent with this finding, Hapli was characterized by large amounts of *nifH*-containing SRB across the soil profile, including *Desulfobacca* and *Desulfovibrio* (Fig. S6B and S7B), which contributed significantly to both the Fe and N cycling indices (Fig. 4C and D). Some sulfur-reducing and sulfur-oxidizing microorganisms have been reported to act as active N_2_ fixers in ecosystems such as sulfidic sediments (31, 66, 67). Obligately anaerobic *Desulfobacca* belonging to *Syntrophaceae* have been identified as major acetate-degrading SRB and common N_2_ fixers in paddy soils (6, 68), and some reports indicate that *Desulfobacca* are aerotolerant and exist in the surface layer of paddy soils (51). In flooded soil, electron acceptors are reduced sequentially according to thermodynamic theory, with oxidative capacity in the order of oxygen > NO_3_^−^ > sulfate and Fe^3+^ oxides (47). Some SRB (e.g., *Desulfovibrio* and *Desulfotomacufum*) are also facultative FeRB or trigger Fe reduction indirectly via sulfide production, a dominant force in Fe biogeochemical cycling (33–35). Bao and Li observed coupling between ferrihydrite reduction and anaerobic ammonium oxidation driven by sulfur redox cycling in paddy soils (14), and a similar process may be involved in the S-Fe-N biogeochemical cycling in Hapli. To obtain a comprehensive understanding of the mechanisms coupling multielement biogeochemical processes in paddy soils, future work should incorporate metagenomic approaches and hydrogeochemical evidence for element transformation (69).

### Conclusions.

The understanding of microbial assembly and biogeochemical processes in the deep subsurface remains limited, especially for different paddy soil types with distinctive redox reactions. Our results provide an integrated perspective on the vertical assembly of soil bacterial and diazotrophic communities in two typical paddy soil types at the local scale. The communities were significantly influenced by soil depth followed by soil type. Compared with the oxidative Hapli stagnic anthrosol, the redoximorphic Fe-accumuli stagnic anthrosol was characterized by a lower Fe cycling index in surface horizons and a lower N cycling index in deep layers. By quantifying the contributions of microbial taxa to the differentiated soil N and Fe cycling indices, our data suggest distinctive biogeochemical processes in the two paddy soil types: Fe-reducing *Geobacter*- and Fe-oxidizing *Rhodanobacter*-mediated Fe-N cycling in Fe-accumuli and sulfur-reducing diazotroph *Desulfobacca*-mediated S-Fe-N cycling in Hapli. These findings further highlight the significant roles played by diazotrophs (e.g., *Geobacter* and *Desulfobacca*) in coupling multiple element cycling processes at the community level.

## MATERIALS AND METHODS

### Soil sampling.

After the rice harvest, paddy soils were sampled from rice fields at nine sites across Anhui Province, China, in October 2015 (Fig. 1A; see also Table S1 in the supplemental material) (maximum distance between two sites of 300 km). The main crop systems were rice-rice, rice-rape, or rice-wheat crop rotation. Details of the sampling sites (e.g., climate and soil properties) are shown in Table S1. At each site, one soil profile (2 m by 4 m, 80- to 120-cm depth) was excavated to the parent material and divided into 3 to 5 horizons according to pedological characteristics. Based on the diagnostic horizons (redoximorphic features and degree of paddy soil development) (15, 40), the nine profiles were classified as two typical types of paddy soil: four as Fe-accumuli stagnic anthrosols and five as Hapli stagnic anthrosols. For each horizon, nine soil cores were randomly collected using soil cutting rings (around the middle zone of each horizon), and every three soil cores were pooled into a composite sample as a replication. A total of 111 soil samples (9 profiles, 3 to 5 horizons for each profile, 3 replications for each horizon) were collected.

Visible plant tissues, stones, and debris were removed from the sampled soils, and each sample was subdivided into three parts. The first portion, which was used for microbial community analysis, was stored at 4°C in the field, transported in a cooler to the laboratory within 36 h, and stored at −80°C before DNA extraction. The second portion was stored at 4°C and was used for the determination of soil pH, moisture, soil nitrate (NO_3_-N), ammonium (NH_4_^+^-N), and available inorganic P content. The third portion was air dried and sieved through a 0.25-mm mesh for the analysis of other soil properties, including soil OM, N, P, K, free Fe oxides, and microelements (Ca, Mg, Fe, Mn, Zn, Cu, and Mo). Detailed methods for determining soil properties are provided in the Text S1.

10.1128/mSystems.01047-21.8 TEXT S1 Determination of soil properties, absolute quantification method for qPCR, and high-throughput sequencing and data analysis. Download Text S1, PDF file, 0.6 MB. Copyright © 2022 Wang et al. 2022 Wang et al. https://creativecommons.org/licenses/by/4.0/ This content is distributed under the terms of the Creative Commons Attribution 4.0 International license.

### DNA extraction, high-throughput sequencing, and qPCR.

Total soil DNA was extracted from 0.5 g of soil using a FastDNA Spin kit for soil (MP Biomedicals LLC, OH, USA) according to the manufacturer’s protocol. The bacterial universal primer pair 515F (5′-GTG CCA GCM GCC GCG G-3′) and 806R (5′-GGA CTA CHV GGG TWT CTA AT-3′), which targets the V4 hypervariable region of the microbial 16S rRNA gene, was used to amplify the total bacterial community (70). The primer pair *nifH*-F (5′-AAA GGY GGW ATC GGY AAR TCC ACC AC-3′) and *nifH*-R (5′-TTG TTS GCS GCR TAC ATS GCC ATC AT-3′) was used to amplify the diazotrophic community (71). PCR amplification and product purification were conducted as described previously (46), and the amplicons were sequenced using the Illumina MiSeq PE300 platform (Majorbio Biotechnology Co., Ltd. Company, Shanghai, China). Quantitative PCR was performed for the total bacterial and diazotrophic populations using an AceQ universal SYBR qPCR master kit (Vazyme Biotech Co., Ltd., Nanjing, China) in a CFX96 optical real-time detection system (Bio-Rad, Laboratories Inc., Hercules, CA, USA). Detailed methods are described in the Text S1.

### Data analysis.

Raw data from Illumina MiSeq high-throughput sequencing were analyzed using the Quantitative Insight into Microbial Ecology (QIIME) 1.9.1 pipeline (12). Low-quality data were removed (sequences with an average quality score of <25 for 16S rRNA gene and <20 for *nifH*). Chimeras were removed using Usearch8 in *de novo* mode. For 16S rRNA gene analysis, sequences were assigned to OTUs at 97% similarity by using UCLUST. Representative sequences for the 16S rRNA gene were aligned by referring to SILVA database release 128 for bacteria at the 80% threshold (41). For diazotrophs, *nifH* sequences were translated into amino acid sequences using the FunGene Pipeline of the Ribosomal Database Project to remove sequences that failed translation using FrameBot (46). Sequences were clustered into OPUs at 95% similarity by using UCLUST. All singleton OTUs/OPUs were deleted. To control the heterogeneity of the number of sequences per sample, each sample of the data set was rarefied to minimal sequencing depth before analyzing the alpha and beta diversity indices. Statistical analysis was conducted using R software (version 3.4.1; R Software for Statistical Computing, Vienna, Austria) with appropriate packages (e.g., vegan, picante, ggcrr, and labdsv; details are listed in the Text S1). Plots were prepared using the package ggplot2 or pheatmap.

### Assessing N and Fe cycling indices.

Soil element cycling indices were quantified using the standardized average of edaphic properties with the multifunc package (72). The N cycling index was calculated using total N, available N, NO_3_-N, and NH_4_^+^-N, and the Fe cycling index was evaluated using total Fe, available Fe, and free Fe oxides (41, 72). For each index, related edaphic properties were normalized (log_10_ transformed as required) and standardized to a common scale using *Z*-score transformation (5).

### Random forest.

Explanatory variables (environmental factors and microbial predictors) for soil type and nutrient cycling indices were predicted by RF analysis with the Random Forest package (12). The importance of each predictor was estimated by the percent increases in the mean square error (MSE) of the explanatory variable between observations and predictions. The significance of the model and cross-validated *R*^2^ values were assessed with 5,000 permutations of the response variables using the rfUtilities package. The significance of predictor importance for the response variables was assessed with the rfPermute package.

### Data availability.

The raw data were deposited in the National Center for Biotechnology Information (NCBI) Sequence Read Archive (SRA) under accession number PRJNA639403 for bacteria and PRJNA639406 for diazotrophs.
