# Supplementary figures and images for: Variations of Bacterial and Diazotrophic Community Assemblies throughout the Soil Profile in Distinct Paddy Soil Types and Their Contributions to Soil Functionality
Source: mSystems. 2022 Mar 1;7(2):e01047-21. doi: 10.1128/msystems.01047-21 (PMC8941939; doi:10.1128/msystems.01047-21)

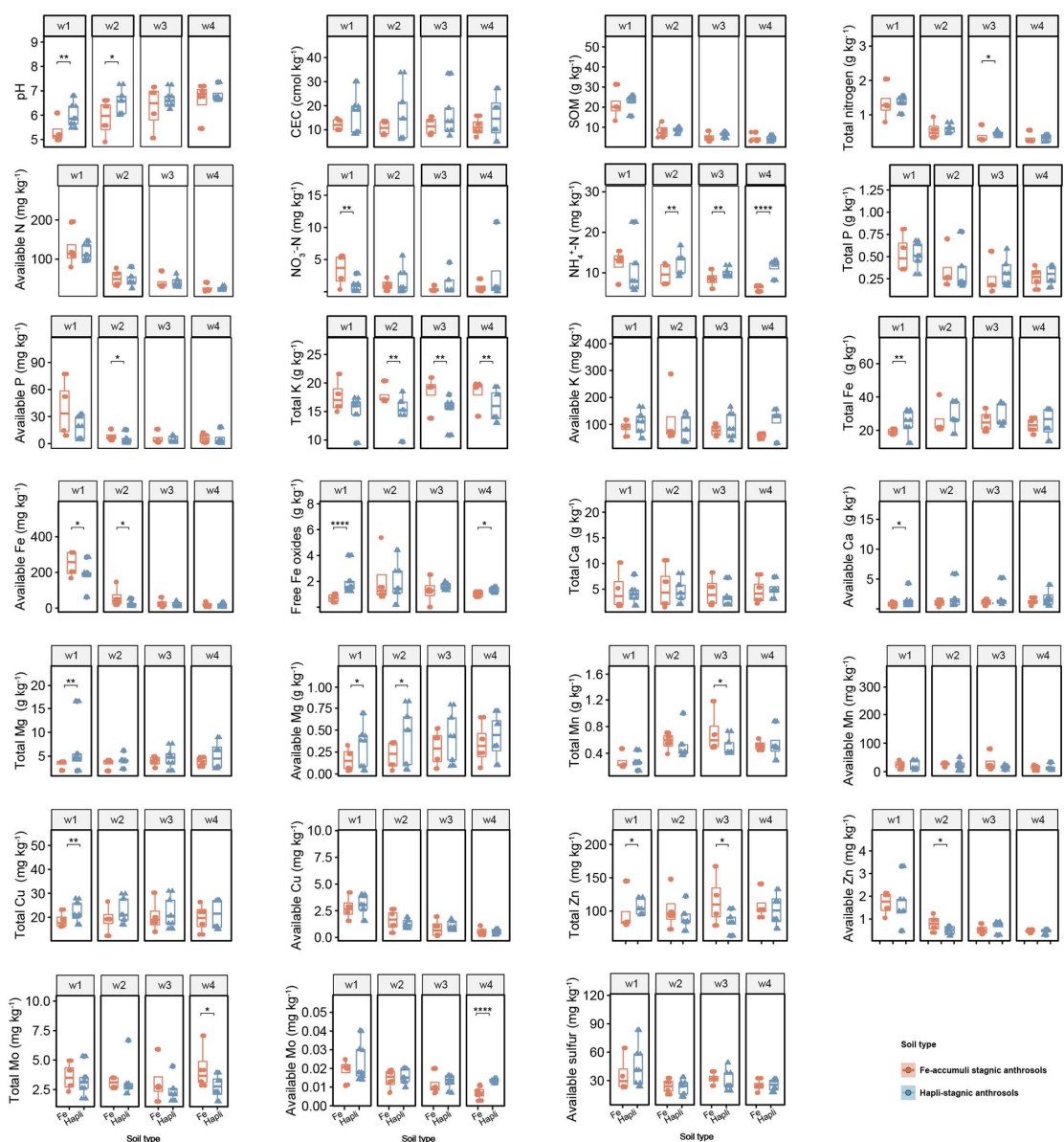

Supplement: FIG S1 [file msystems.01047-21-sf001.pdf]

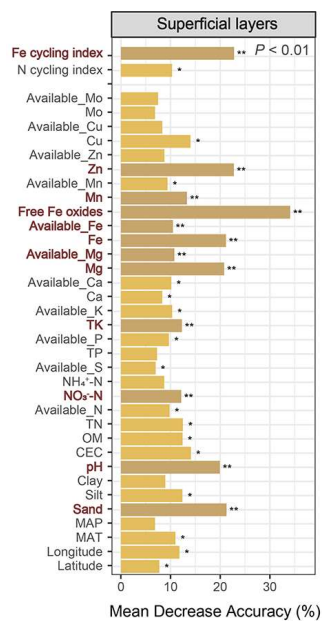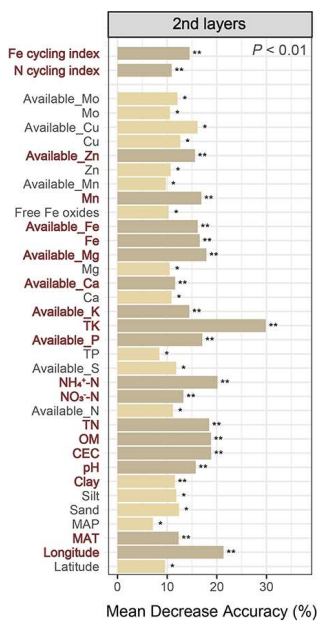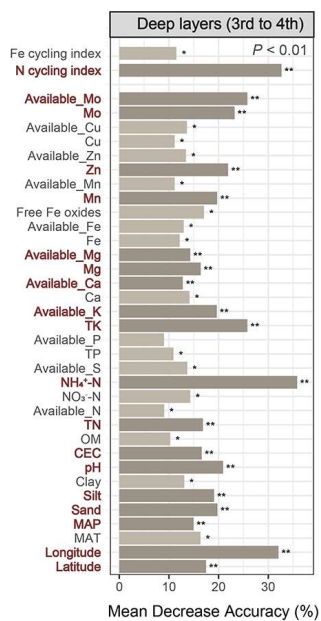

Supplement: FIG S2 [file msystems.01047-21-sf002.pdf]

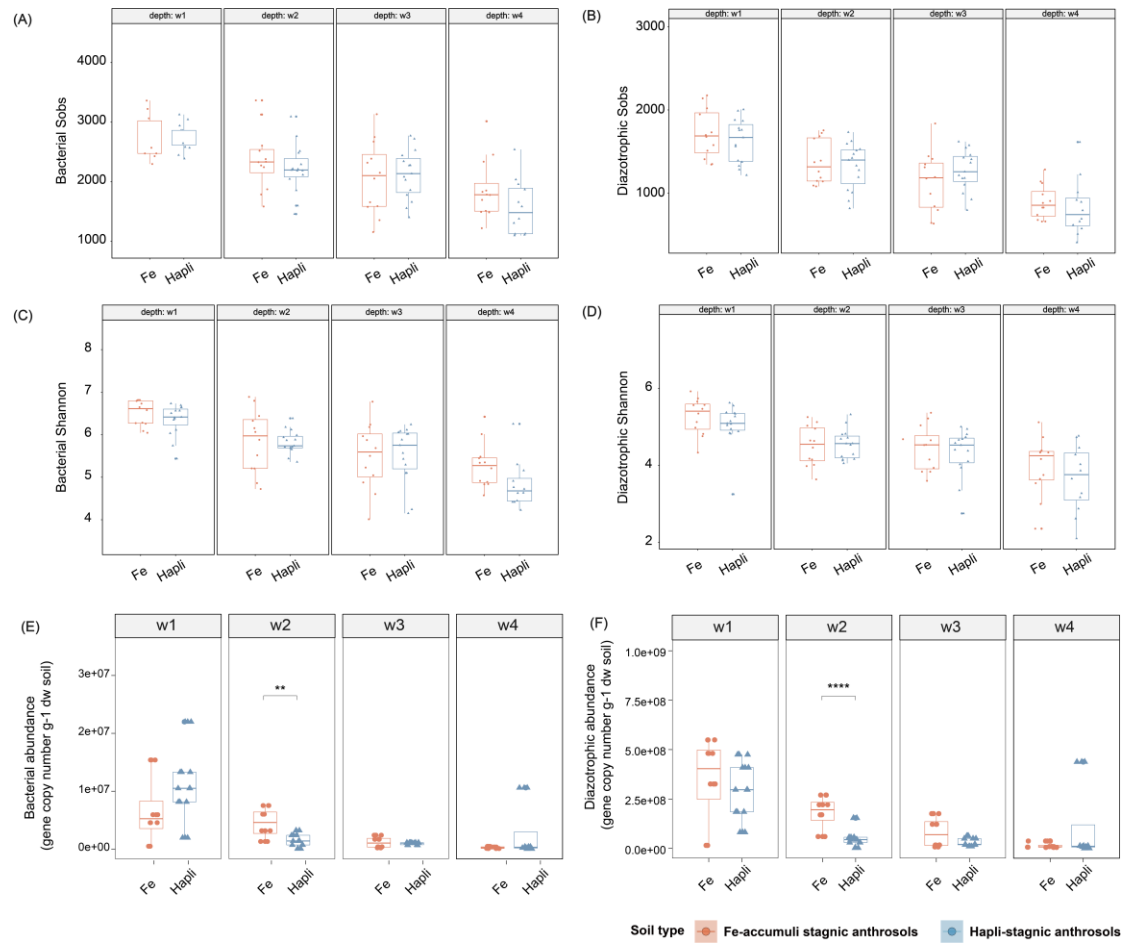

Supplement: FIG S3 [file msystems.01047-21-sf003.pdf]

(A) Fe-accumuli stagnic anthrosols

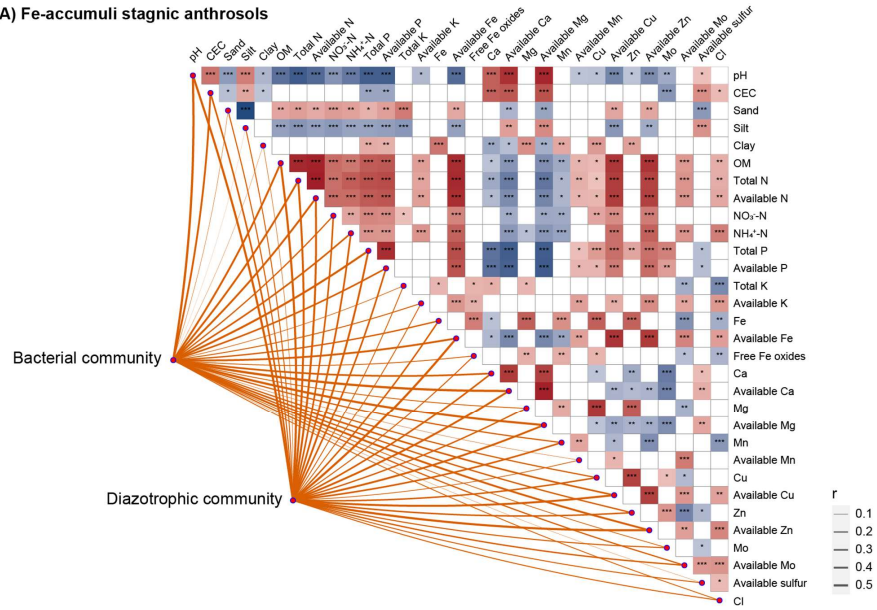

(B) Hapli-stagnic anthrosols

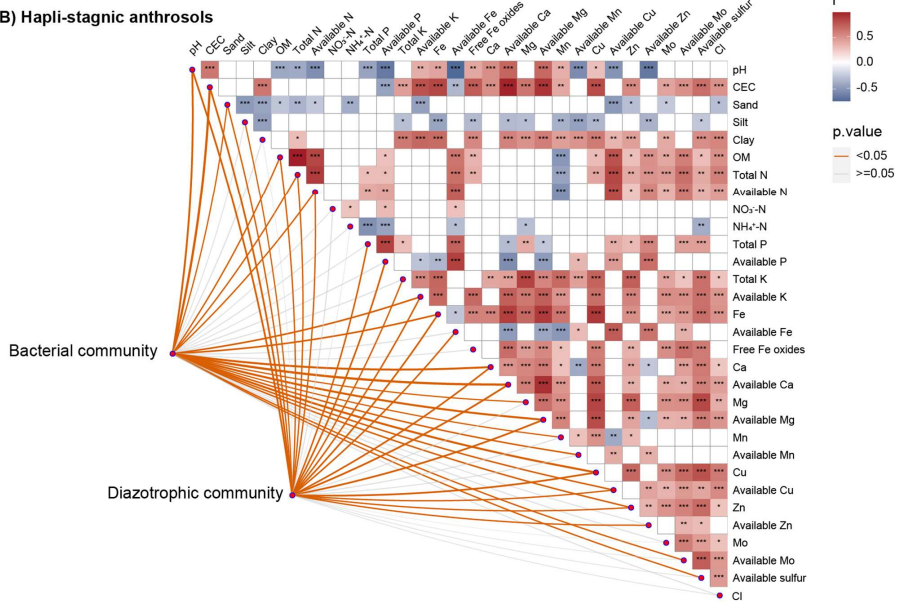

Supplement: FIG S5 [file msystems.01047-21-sf005.pdf]

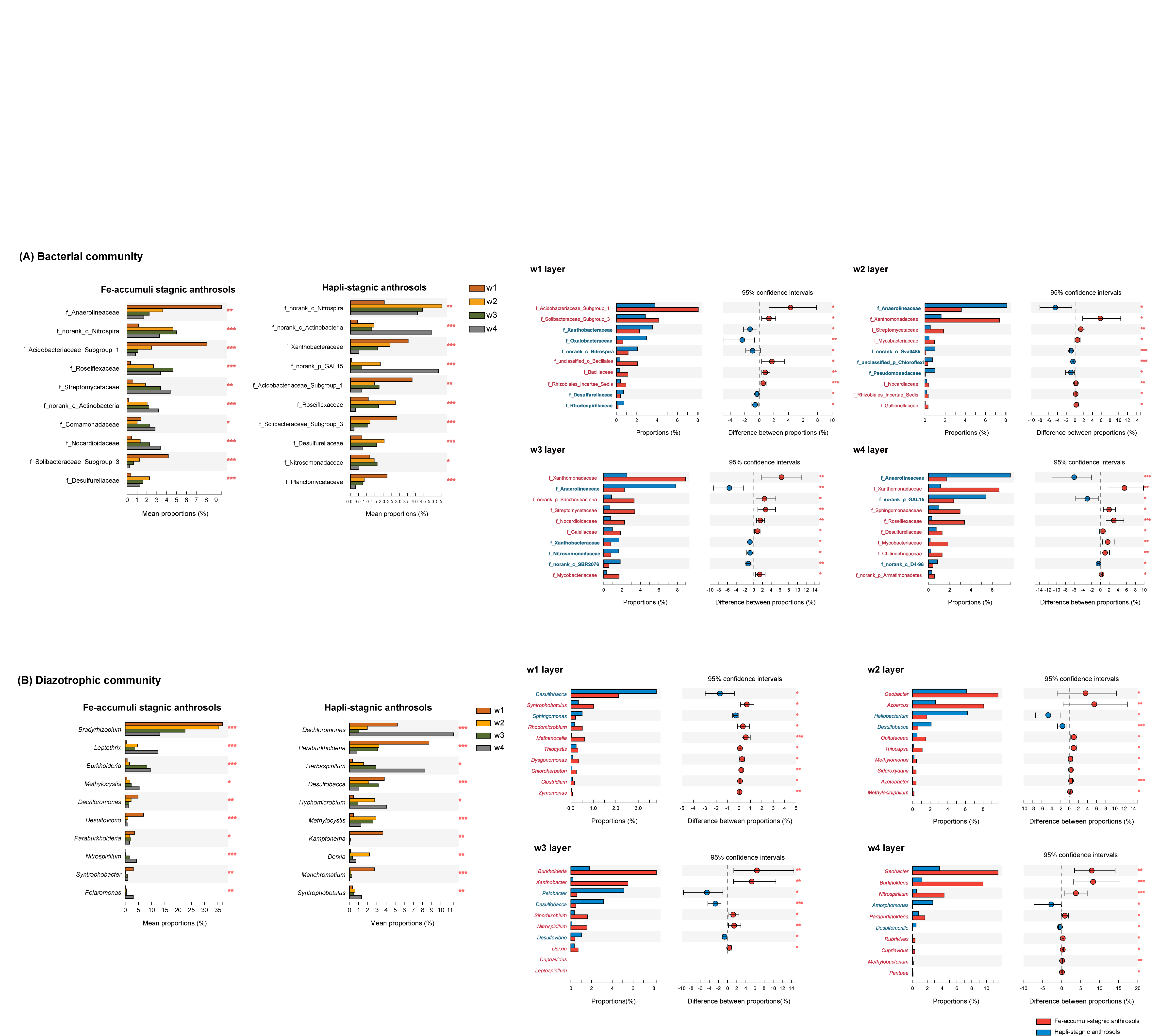

Supplement: FIG S6 [file msystems.01047-21-sf006.tif]

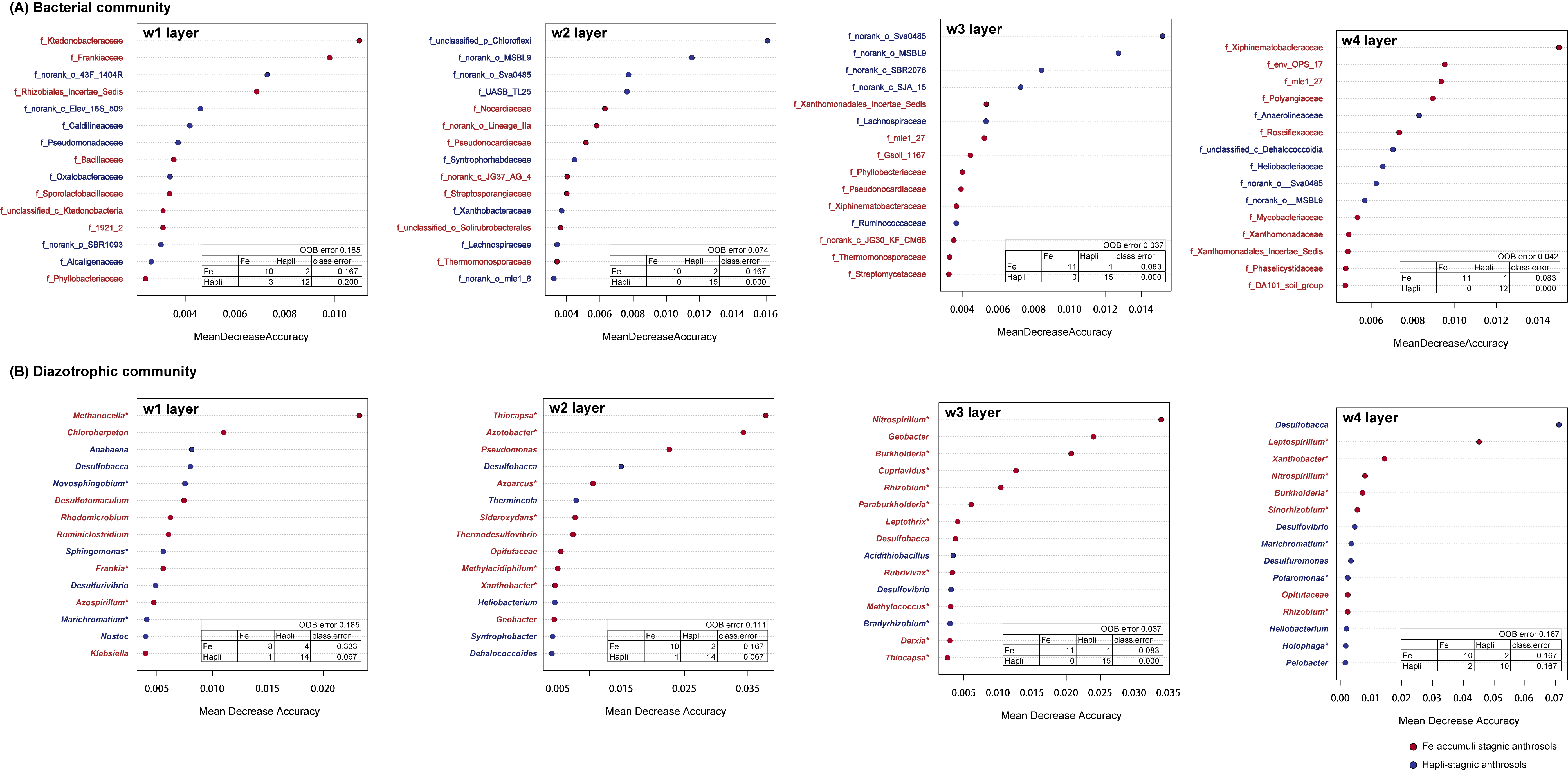

Supplement: FIG S7 [file msystems.01047-21-sf007.tif]
