# Supplementary material for: Variations of Bacterial and Diazotrophic Community Assemblies throughout the Soil Profile in Distinct Paddy Soil Types and Their Contributions to Soil Functionality
Source: mSystems. 2022 Mar 1;7(2):e01047-21. doi: 10.1128/msystems.01047-21 (PMC8941939; doi:10.1128/msystems.01047-21)
Supplement: FIG S4 [file msystems.01047-21-sf004.pdf]

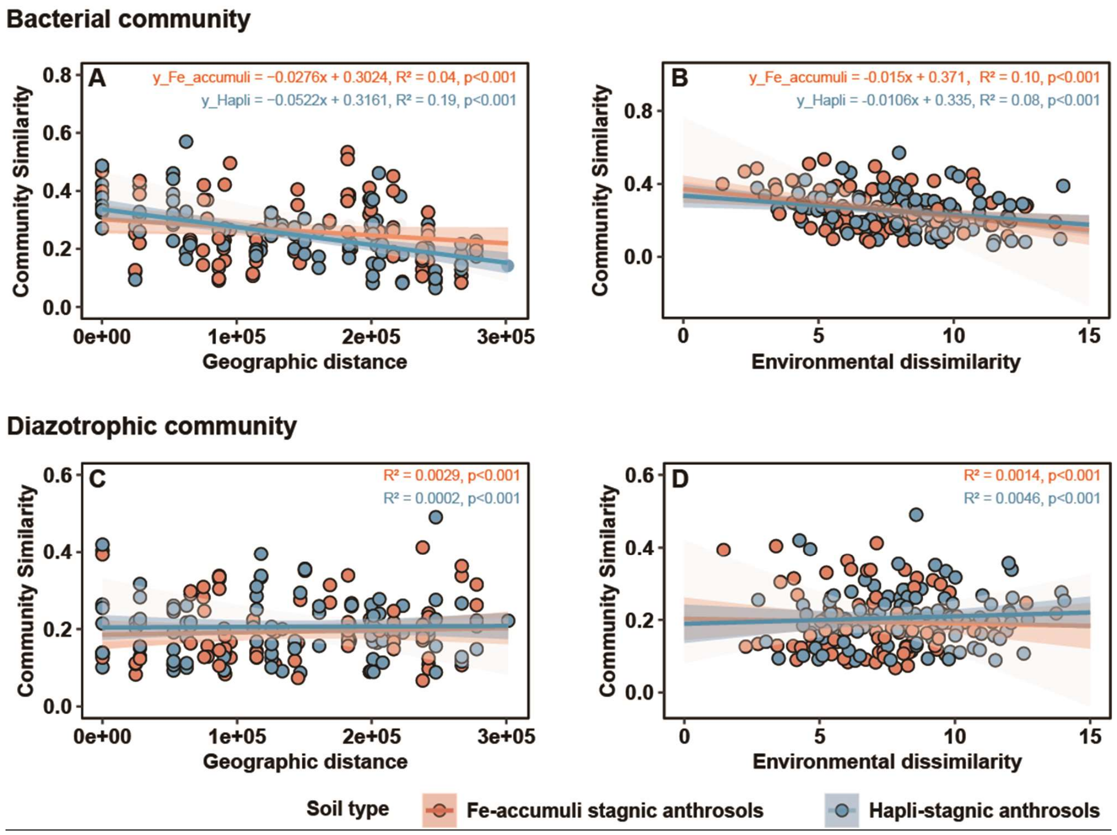
Bacterial community
A
y_Fe_accumuli = −0.0276x + 0.3024, R² = 0.04, p<0.001
y_Hapli = −0.0522x + 0.3161, R² = 0.19, p<0.001
Community Similarity
0.8
0.6
0.4
0.2
0.0
0e+00
1e+05
2e+05
3e+05
Geographic distance
B
y_Fe_accumuli = -0.015x + 0.371, R² = 0.10, p<0.001
y_Hapli = -0.0106x + 0.335, R² = 0.08, p<0.001
Community Similarity
0.8
0.4
0.0
0
5
10
15
Environmental dissimilarity
Diazotrophic community
C
R² = 0.0029, p<0.001
R² = 0.0002, p<0.001
Community Similarity
0.6
0.4
0.2
0.0
0e+00
1e+05
2e+05
3e+05
Geographic distance
D
R² = 0.0014, p<0.001
R² = 0.0046, p<0.001
Community Similarity
0.6
0.4
0.2
0.0
0
5
10
15
Environmental dissimilarity
Soil type
Fe-accumuli stagnic anthrosols
Hapli-stagnic anthrosols
