# Supplementary material for: Variations of Bacterial and Diazotrophic Community Assemblies throughout the Soil Profile in Distinct Paddy Soil Types and Their Contributions to Soil Functionality
Source: mSystems. 2022 Mar 1;7(2):e01047-21. doi: 10.1128/msystems.01047-21 (PMC8941939; doi:10.1128/msystems.01047-21)
Supplement: TEXT S1 [file msystems.01047-21-s0001.pdf]

Supplementary texts for methods

Soil properties

Soil physiochemical properties were measured according to previous studies (Fan et al., 2019). Soil pH was measured in a soil suspension with a fresh soil to water ratio of 1:5 (shaking for 30 min) using a pH meter (E20-FiveEasy TM pH, Mettler Toledo, German). Soil moisture content (%) was determined by calculating the percentage of the fresh weight loss after oven drying. Soil particle size distribution was determined by the hydrometer method (Bouyoucos, 1962). Soil organic matter and total nitrogen (N) were analyzed using rapid dichromate oxidation-titration method and Kjeldahl procedure, respectively. Nitrate (NO_3^--N), ammonium (NH_4^+-N), and available N were extracted with 5.0 g fresh soil in 50 mL 2 M KCl by shaking. The supernatant were filtered and analyzed with a continuous flow analytical system (San^{++} system, Skalar, Holland). Phosphorus (P) and potassium (K) were measured using the molybdenum-antimony (Mo-Sb) colorimetry method and flame spectrophotometry method (FP640, INASA, China), respectively.

The quantities of each total microelement (Ca, Mg, Fe, Mn, Zn, Cu and Mo) were measured by inductively coupled plasma atomic emission spectrophotometer (ICP-AES), following digestion by HF-HNO_3-$HClO_4$ (Wang et al., 2019). Available microelements were analyzed using the inductively coupled plasma mass spectrometry with high performance liquid chromatography after digestion with diethylene triamine pentacetic acid (Wang et al., 2019).

Absolute quantification method for qPCR

Total soil DNA was extracted from 0.5 g of soil using a FastDNA® SPIN Kit for soil (MP Biomedicals LLC, Ohio, USA), according to the manufacturer`s protocol. The concentration of total soil DNA and purity were measured by a Nanodrop ND-1000 spectrophotometer (NanoDrop Technologies Inc., Wilmington, DE, USA) and stored at −20°C for further analysis.

For quantitative polymerase chain reaction (qPCR), the standard curve was constructed using a 10-fold serially-diluted plasmid DNA for each target gene. The tagged genes of bacterial and diazotrophic communities were amplified using primer pairs 515F/806R (5'- GTGCCAGCMGCCGCGG-3')/(5'- GGACTACHVGGGTWTCTAAT-3') and nifH-F/nifH-R (5'-AAAGGYGGWATCGGYAARTCCACCAC-3')/(5'-TTGTTSGCSGCRTACATSGCCATCAT-3'), respectively. The amplication mixture of 20 μL contained 10 μL SYBR qPCR Master Mix, 0.4 μL of each primer, and 1 μL template DNA. The thermocycling conditions for target genes were based on references(Shuo et al., 2018; Fan et al., 2019). Sterilized deionized water was used as template in negative control. Three technical replications were carried out for each DNA sample. The amplification efficiency was 98.2-106.7% with an R^2 value of 0.990-0.996. Agarose gel electrophoresis and melting curve analysis were used to check the specificity of the amplification products. The values and their standard deviations (SD) were based on triplicate samples.

High throughput sequencing

The PCR amplification system was conducted based on Fan et al. (2020)(Fan et al., 2019). A unique 12 bp-barcode was added at the 5'-end of the reverse primer of each sample. PCR reaction was carried out at 95°C for 3 min, then 30~35 cycles of 95°C for 30 s, 55°C for 30 s, 72 °C for 45 s, followed by final extension at 72°C for 10 min. After detection by agarose gel electrophoresis (w/v, 2%) and purification, triplicate PCR products for each sample were pooled together at equal molar amounts for library construction. The PCR amplicons were sequenced using the platform of Illumina MiSeq PE300 (Majorbio Biotechnology Co., Ltd Company, Shanghai, China). Raw data were deposited under the National Centre for Biotechnology Information (NCBI) Sequence Read Archive (SRA) with accession number PRJNA639403 for bacteria and PRJNA639406 for diazotrophs.

Data analysis

Across 111 paddy soil samples, a total of 5,604,253 and 2,267,904 high-quality bacterial and diazotrophic sequences were obtained, which grouped into 6,797 OTUs and 5,434 OPUs, respectively. Bioinformatics analysis were conducted using R software (Version 3.4.1; R Software for Statistical Computing, Vienna, Austria). Partial least squares discriminant analysis (PLS-DA) was used to evaluate the impacts of soil types and profiles on the Bray-Curtis distances of microbial community composition (‘*mixOmics*’ package). ANOSIM/ADONIS (an analog of univariate ANOVA) with the

Spearman rank correlation method were employed to statistically evaluate community variances among groups (‘*vegan*’ package). The influence of soil types and soil depths on soil properties, microbial gene copy numbers and microbial community structure were analyzed with Two-way analysis of variance (ANOVA) and PERMANOVA analysis using ‘*vegan’* packages(Mapelli et al., 2018).

Correlations between soil physicochemical factors and microbial community composition turnover were calculated using a Mantel test. A partial Mantel test was conducted to examine the relative impacts of geographic distance and environmental factors on community composition (‘*vegan*’ package). The distance-decay relationship was estimated based on linear regression with compositional community similarity, and geographic/environmental distance. Geographic distance was calculated using a matrix of pairwise distance among each site (‘*vegan*’ and ‘*geosphere*’ package)(Fan et al., 2019). Compositional similarity was calculated using the Bray-Curtis distance, and environmental distance was calculated using the soil properties based on the Euclidean distance.

Spearman's rho correlation analysis was used to calculate the effect of environmental factors on microbial features using ‘*psych’* packages. Significant differences between two groups were analyzed by Wilcoxon rank sum test, and variance of multiple groups by Kruskal-Wallis test, with false discovery rate (FDR) adjusted *P* value using ‘*ggpubr*’ package.

References

Bouyoucos, G.J., 1962. Hydrometer method improved for making particle size analyses of soils. Agronomy Journal 54, 464-465. doi:

Fan, K.K., Delgado-Baquerizo, M., Guo, X.S., Wang, D.Z., Chu, H.Y., 2019. Suppressed N fixation and diazotrophs after four decades of fertilization. Microbiome 7, 143. doi: 10.1186/s40168-019-0757-8

Hardy, O., 2008. Testing the spatial phylogenetic structure of local communities: statistical performances of different null models and test statistics on a locally neutral community. Journal of Ecology 96, 914-926. doi:

Mapelli, F., Marasco, R., Fusi, M., Scaglia, B., Daffonchio, D., 2018. The stage of soil development modulates rhizosphere effect along a High Arctic desert chronosequence. Isme Journal 12, 1188–1198. doi: 10.1038/s41396-017-0026-4

Purcell, D., Sompong, U., Yim, L.C., Barraclough, T.G., Peerapornpisal, Y., Pointing, S.B., 2007. The effects of temperature, pH and sulphide on the community structure of hyperthermophilic streamers in hot springs of northern Thailand. FEMS Microbiology Ecology 60, 456–466. doi:

Shuo, J., Chen, W.M., Wang, J.L., Du, N.N., Li, Q.P., 2018. Soil microbiomes with distinct assemblies through vertical soil profiles drive the cycling of multiple nutrients in reforested ecosystems. Microbiome 6, 146. doi: 10.1186/s40168-018-0526-0

Wang, X.J., Liu, B., Ma, J., Zhang, Y., Hu, T., Zhang, H., Feng, Y., Pan, H., Xu, Z., Liu, G., Lin, X., Zhu, J., Bei, Q., Xie, Z.B., 2019. Soil aluminum oxides determine biological nitrogen fixation and diazotrophic communities across major types of paddy soils in China. Soil Biology & Biochemistry 131 81–89. doi: 10.1016/j.soilbio.2018.12.028
